# Accuracy of a Custom Physical Activity and Knee Angle Measurement Sensor System for Patients with Neuromuscular Disorders and Gait Abnormalities

**DOI:** 10.3390/s150510734

**Published:** 2015-05-06

**Authors:** Frank Feldhege, Anett Mau-Moeller, Tobias Lindner, Albert Hein, Andreas Markschies, Uwe Klaus Zettl, Rainer Bader

**Affiliations:** 1 Department of Orthopaedics, University Medicine Rostock, Doberaner Str. 142, 18057 Rostock, Germany; E-Mails: anett.mau-moeller@med.uni-rostock.de (A.M.-M.); tobias.lindner@med.uni-rostock.de (T.L.); rainer.bader@med.uni-rostock.de (R.B.); 2 Institute of Computer Science, University of Rostock, Albert-Einstein-Str. 22, Rostock 18059, Germany; E-Mail: albert.hein@uni-rostock.de; 3 Medizintechnik Rostock GmbH, Zur Himmelspforte 1, Rostock 18055, Germany; E-Mail: a.markschies@mtronline.de; 4 Department of Neurology, University Medicine Rostock, Gehlsheimer Str. 20, Rostock 18147, Germany; E-Mail: uwe.zettl@med.uni-rostock.de

**Keywords:** physical activity, accelerometer, activity assessment, knee motion, wearable sensor system, range of motion measurement

## Abstract

Long-term assessment of ambulatory behavior and joint motion are valuable tools for the evaluation of therapy effectiveness in patients with neuromuscular disorders and gait abnormalities. Even though there are several tools available to quantify ambulatory behavior in a home environment, reliable measurement of joint motion is still limited to laboratory tests. The aim of this study was to develop and evaluate a novel inertial sensor system for ambulatory behavior and joint motion measurement in the everyday environment. An algorithm for behavior classification, step detection, and knee angle calculation was developed. The validation protocol consisted of simulated daily activities in a laboratory environment. The tests were performed with ten healthy subjects and eleven patients with multiple sclerosis. Activity classification showed comparable performance to commercially available activPAL sensors. Step detection with our sensor system was more accurate. The calculated flexion-extension angle of the knee joint showed a root mean square error of less than 5° compared with results obtained using an electro-mechanical goniometer. This new system combines ambulatory behavior assessment and knee angle measurement for long-term measurement periods in a home environment. The wearable sensor system demonstrated high validity for behavior classification and knee joint angle measurement in a laboratory setting.

## 1. Introduction

Physical activity (PA) has been defined as any “bodily movement produced by skeletal muscles which results in energy expenditure” [1]. Over the last three decades, PA has become a widely used evaluation criterion for quality of life and general health assessments as well as the effectiveness of therapy for different diseases [2,3,4,5,6,7]. Therefore, a variety of diagnostic tools, including questionnaires, self-report diaries, pedometers, heart frequency monitors, accelerometers, and the doubly-labeled water method (DLW), have been developed and used, and there have been different claims regarding their accuracy, validity, and ease of use [8].

Since accelerometer-based sensors have become reasonably small, several smart, portable devices for the assessment of ambulatory behavior (AB) as a measure for PA have been introduced [8,9,10]. Several devices with different ranges of functions, outcome measures, and specific patient body-positioning requirements, which are available on the market, have been evaluated [8,9,11,12].

While AB assessment is already being used for long-term measurements in the home environment [13,14,15], gait analysis (GA), which requires expensive, motion-capturing systems is usually performed in a laboratory [16,17,18]. However, less complex methods that utilize combinations of wearable inertial sensors [19,20,21,22,23], wearable ground reaction force sensors [24,25] or other sensors [26,27] for the assessment of single joint movements and/or spatiotemporal gait parameters outside of specialized gait laboratories have been developed recently.

Because the long-term assessment of AB in combination with joint kinematics may deliver meaningful data about functional outcomes and rehabilitation status in orthopedic and neuromuscular research [28], our system integrates gait assessments at the “macro level” [29] (AB) and “micro level” [29] (GA) into one wearable device. To our knowledge, it is one the first systems that opens up the possibility of continuous kinematic analysis of joint motion in combination with data for the ambulatory activities in the participant’s everyday environment, and it might provide more generalizable results compared with GA at specific time points in a controlled laboratory environment or AB alone [29,30].

Therefore, the aim of the present study was to evaluate this newly developed, small, and low-power-consuming measurement system in healthy subjects and patients with multiple sclerosis (MS) in the home environment.

## 2. Methods

### 2.1. Instrumentation

**Figure 1:**
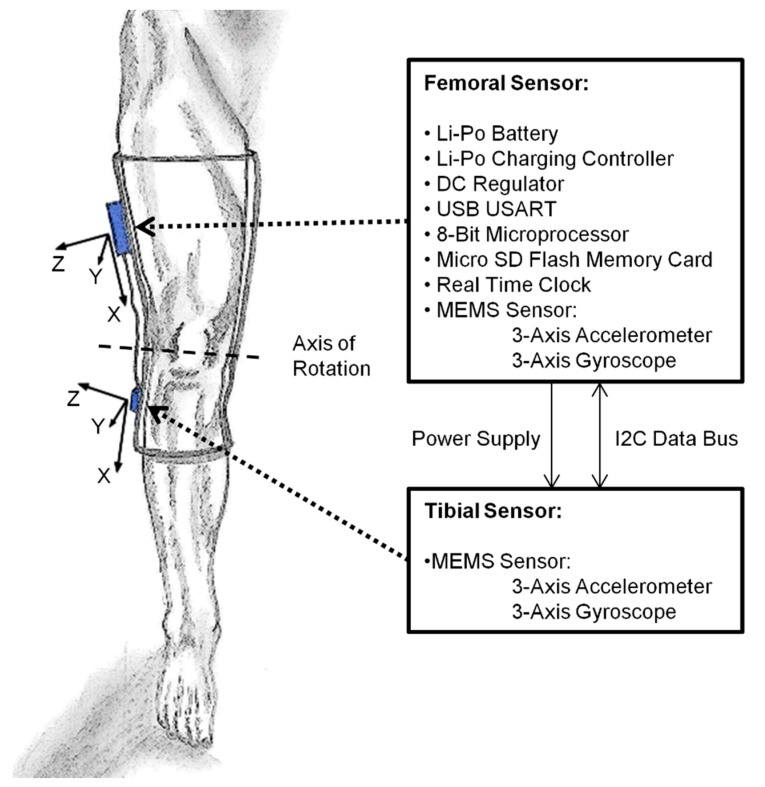
Schematic illustration of the sensor and monitoring system integrated into an orthosis.

The measurement system consists of five core components: a microprocessor, inertial sensors, memory, a battery, and a charging circuit. We used an 8-bit RISC Microprocessor (Atmega328; Atmel; San Jose, CA, USA) to control our system. To measure the inertial data, we chose two microelectromechanical systems (MEMS) chips (MPU-6050; InvenSense Inc.; San Jose, CA, USA), each of which incorporates a digital triaxial accelerometer and a digital triaxial gyroscope. For timing processes, we used a real time clock (RV-8564-C2; Micro Crystal Switzerland; Grenchen, Switzerland). The raw data is stored in plain text files on a micro SD card. The microcontroller, the two MEMS sensors, and the real time clock are connected with an I2C bus. The SD card is interfaced with an SPI bus. The system also contains a USB-UART IC (FT232RL; Future Technology Devices International Limited; Glasgow, UK) for a PC connection and configuration of the sensor system. It is powered by a small, lithium polymer battery, which can be recharged using a USB connector with a small charging controller (MCP73831/2; Microchip Technology Inc.; Chandler, AZ, USA). Battery runtime and system functionality were tested in healthy subjects in long-term recordings, which are not further described in this study. With a fully charged battery (3.7 Wh), the sensor system can operate continuously for about 4–5 days. Prototypes of our system consisting of separate breakout boards are fitted into two small casings. The sensors are attached laterally at the thigh and shank, as shown in Figure 1, approximately five to ten centimeters from the knee joint line.

The tibial sensor is separately contained in a 56 × 20 × 12 mm housing. The rest of the system, including the battery, is stored inside an 80 × 40 × 15 mm casing. The tibial sensor is connected to the femoral system with a thin flexible wire containing leads for the I2C bus and the power supply. The overall weight of the sensor system is about 65 g. The system stores the raw movement data at a sample rate of 50 Hz. Each data block consists of 12 signals containing both triaxial accelerometer values and triaxial gyroscope values at 16-bit resolution. Seven days of recording will generate about 2 GB of raw data. The memory capacity of the system is defined by the selected micro SD card size. Memory cards up to 16 GB have been tested. Recorded movement data is transferred to a personal computer after the measurement is finished. Dedicated software is used to categorize and evaluate the ambulatory behavior and joint angle data.

### 2.2. Software Algorithm

Currently, all data processing is done off-line on a personal computer. The algorithm was designed to be as simple as possible so that at least parts of it can be programmed directly onto the microprocessor in the future. In this way, the data could be calculated directly by the sensor system itself, which allows only the results to be stored, thereby minimizing the overall data output. Using this approach, the post-processing times for long-term measurements can be reduced significantly. The proposed algorithm classifies the measured activity into four categories: lying, sitting, standing, and walking in a second-by-second scheme. The algorithm detects and counts the steps taken while walking. To evaluate the patient’s range of motion, the software calculates the flexion/extension angle of the knee joint by combining the accelerometer and gyroscope data. The software algorithm was programmed using LabView 2009 (National Instruments; Austin, TX, USA). It consists of the following parts: raw data filtering and frequency separation, knee angle calculation, activity classification, and step detection. The parts are described in detail below.

#### 2.2.1. Raw Data Filtering and Frequency Separation

**Figure 2:**
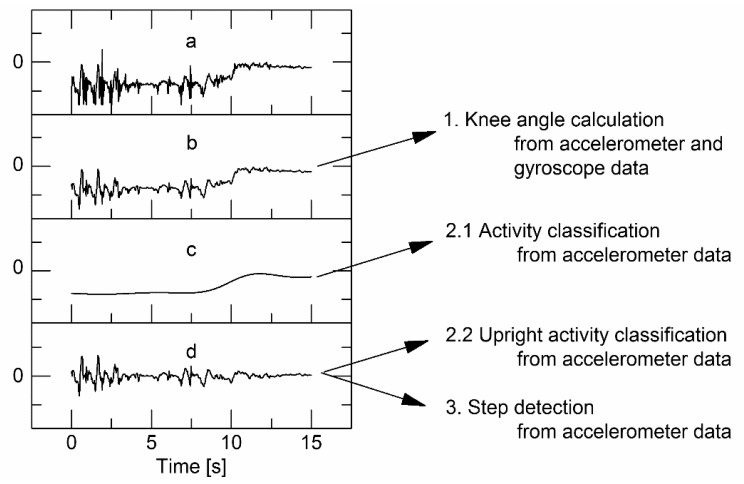
Signal filtering steps; (**a**) raw signal; (**b)** 20 Hz lowpass filtered (*S_20Hz_*); (**c**) gravitation based component (*S_grav_*); 0.2 Hz lowpass filtered; (**d**) movement based component (*S_mov_*).

We used a third-order elliptical infinite impulse response (IIR) low-pass filter shown in Equation (1) (cutoff frequency 20 Hz, passband ripple 0.01 dB, stopband −100 dB) to remove higher frequency components and noise from all sensor signals (*S_20Hz_,* Figure 2b) [31], as all relevant human body movements are expected to be below 15 Hz [32]. Gravitational- and movement-based parts of the accelerometer signals are separated using a second filtering step. Thereafter, another third-order elliptical IIR low-pass filter shown in Equation (1) (cutoff frequency 0.2 Hz, passband ripple 0.01 dB, stopband −100 dB) is applied to *S_20Hz_*, resulting in an accelerometer signal that only contains gravitation (posture)-based components (*S_grav_*, Figure 2c) [31]. A signal containing just the movement-based parts (*S_mov_*) is calculated as the linear difference between the original signal and the gravitational signal: $S_{mov}=S_{20Hz}-S_{grav}$ (Figure 2d) [31]. The IIR filter coefficients (b_0_ … b_3_, a_1_ … a_3_) for all elliptical low-pass filters were calculated using MATLAB 2013 (MathWorks; Natick, MA, USA):

(1) $$y(t)=b_{0}x(t)+b_{1}x(t-1)+b_{2}x(t-2)+b_{3}x(t-3)+a_{1}y(t-1)+a_{2}y(t-2)+a_{3}y(t-3)$$

#### 2.2.2. Knee Angle Calculation

Our approach approximates the human knee as a hinge joint. For optimal results, the *z*-axes of both sensors and the main axis of knee rotation will need to coincide. As this is not easily achievable in human anatomy (Figure 1), the precision of the calculated knee angle will be reduced [22]. Godfrey *et al*. proposed the use of the scalar product for the tilt estimation of a chest-mounted accelerometer sensor [33]. Our algorithm uses a comparable approach for the calculation of the knee angle and combines that result with the gyroscope data using a complementary filter [34]. The knee flexion-extension angle is calculated as the scalar product from both sensors’ accelerometer data vectors (*S_20Hz_*), defined in Equations (2) and (3) in the sagittal plane. The rotational direction is calculated using the vector product shown in Equation (4). This angle is then passed through a third-order elliptical IIR filter, Equation (1) (cutoff frequency 2 Hz, passband ripple 0.01 dB, stopband −100 dB) to reduce shock and vibration influences. A complementary filter which is described in Equation (6) is used to combine the resulting low-pass filtered angle from the accelerometer sensors with the angular velocity from the gyroscope sensors (*S_20Hz_*) described in Equation (5), which are sensitive to signal drift:

(2) $$acc_{f}=\left[\begin{array}{ccc} acc_{f,x} & acc_{f,y} & acc_{f,z} \end{array}\right]$$

(3) $$acc_{t}=\left[\begin{array}{ccc} acc_{t,x} & acc_{t,y} & acc_{t,z} \end{array}\right]$$

(4) $$\alpha_{acc}(acc_{f},acc_{t})=\{\begin{array}{cc} \cos^{-1}\frac{acc_{f}\cdot acc_{t}}{\left|acc_{f}\right|\cdot \left|acc_{t}\right|} & {\left(acc_{f}\times acc_{t}\right)}_{z}<0\\ -\cos^{-1}\frac{acc_{f}\cdot acc_{t}}{\left|acc_{f}\right|\cdot \left|acc_{t}\right|} & {\left(acc_{f}\times acc_{t}\right)}_{z}\geq 0 \end{array}$$

(5) $$\omega_{gyr}=\omega_{gyr,\;f}-\omega_{gyr,\;t}$$

(6) $$\alpha (t)=0.93\cdot (\alpha (t-1)+\omega_{gyr}\cdot dt)+0.07\cdot \alpha_{acc}(t)$$

The complementary filter coefficients were determined empirically. If the calculated knee angle shows a deviation from a known knee angle at a defined posture (*i.e.*, standing upright) due to sensor misalignment, it can be calibrated by adding individual offsets for thigh and shank sensor orientations.

#### 2.2.3. Activity Classification

The activity classification algorithm is based on a threshold detection method of each sensor’s orientation compared to the gravity vector, thereby resulting in low computational complexity. Comparable algorithms using single or multiple accelerometer sensors attached to the body have been presented by Karantonis *et al*. [31], Lyons *et al*. [35], and Culhane *et al*. [36].

The posture based part of the signal (*S_grav_*) is used to separate the lying, sitting, and upright activity classes [31]. The two-dimensional angle between each sensor’s accelerometer output vector and the vector representing its upright vertical position in the sagittal plane is calculated. This tilting angle is compared to an angular threshold of 45°. If a sensor is tilted more than 45° in the sagittal plane, it is defined as being horizontal, otherwise it is defined as being vertical. Additionally, the activity is categorized as lying when one or both sensors are tilted sideways by more than 45° in the coronal plane. Correction of sensor misalignment is performed using the calibration offsets for the thigh and shank orientations as described in Section 2.2.2. The activity is selected using the decision scheme shown in Table 1 for each data sample block.

**Table 1:** Decision table for activity classification.

		Tibial Sensor		
		Horizontal	Vertical	Sideways
**Femoral Sensor**	**Horizontal**	lying	sitting	lying
	**Vertical**	undefined	upright activity	lying
	**Sideways**	lying	lying	lying

A majority voting method is used to reduce the 50 Hz classification rate into a second-by-second classification scheme. The actual activity for each second is selected as the activity class with the most assigned samples from that second. Upright activity is then further separated into standing and walking activity by intensity evaluation of the movement-based signal parts (*S_mov_*). The movement intensity is calculated as the normalized signal magnitude area (SMA) [31] by summarizing the absolute value of all six accelerometer axes from each data sample block, averaging the results for each second, and scaling the result to a multiple of the mean gravitational acceleration (g = 9.81 m/s²), which is shown in Equation (7). The scaling factor (gain) can be calculated from the digital sensor’s range and resolution or measured in a steady sensor position and assumed to be constant. If the SMA value exceeds an adaptive threshold, the activity is classified as walking. The adaptive threshold is calculated every 30 s of walking activity by averaging the corresponding 30 SMA values from those non-overlapping windows. The result is scaled by a factor of 0.5, Equation (8) [37]. The initial threshold for detecting the first 30 s of walking is set to a value of 0.5 g which was selected empirically. This variable threshold enables the algorithm to adapt to different intensities of patient activity:

(7) $$SMA=\left(\frac{1}{50}\sum_{t=1}^{50}\sum_{i=1}^{6}\left|S_{mov,\;i}(t)\right|\right)\cdot gain$$

(8) $$Th_{walk}=\frac{1}{30}\cdot \sum_{t=1}^{30}SMA_{walk}(t)\cdot 0.5$$

#### 2.2.4. Step Detection

We chose the *x*-axis signal from the tibial accelerometer sensor for our step detection algorithm, as it showed the most distinctive peaks in a manual signal evaluation of walking. The step detection algorithm is based on a template-matching method described by Ying *et al*. [38]. This method is described in Equation (9) and compares a moving window of continuous signal data to a predefined step template by evaluating the normalized cross-correlation between them. The result for an identical signal and template is 1. Every time a peak in the continuous calculated correlation coefficient exceeds a predefined threshold of 0.4, the maximum peak value in the corresponding window of the sensor data is searched for and its position is marked as a step. Every time the detected step shows a correlation coefficient below a second threshold of 0.6, a new template is generated by calculating a weighted average of the previous template and the new sensor data shown in Equation (10), thereby resulting in an algorithm that adapts to changes in gait. The first template is generated by averaging the signal windows from ten steps detected using the Pan-Tompkins method for peak detection, which was originally developed for the detection of the R Peak in electrocardiography (ECG) signal analysis [39] and was successfully used for step detection by Ying *et al*. [38] and Marschollek *et al*. [40]:

(9) $$R_{N}(k)=\frac{acc_{t,\;x}\cdot template}{\sqrt{\left|acc_{t,\;x}\right|\cdot \left|template\right|}}$$

(10) $$template_{new}=0.9\cdot template_{old}+0.1\cdot acc_{t,\;x}$$

### 2.3. Algorithm and Sensor Evaluation

System testing and validation were performed on two groups consisting of 10 healthy volunteers (Group A, nine males, one female; age 30.4 ± 7.7 years; weight 74.2 ± 13.8 kg; height 1.79 ± 0.09 m) and 11 participants suffering from multiple sclerosis (MS) that were preselected with an Expanded Disability Status Scale (EDSS) score between 3 and 6 (Group B, seven males, four females; age 49.5 ± 7.4 years; weight 77.3 ± 18.6 kg; height 1.7 ± 0.1 m; EDSS 4.6 ± 1.1). The study was conducted according to the Declaration of Helsinki and approved by the local ethics committee (Registration No. A 2012-0096). Prior to participation, informed written consent was obtained from all subjects. The tests were performed in a laboratory, and the subjects were asked to execute different predefined tasks simulating daily activities in sequential order. The exercises themselves were not constrained to specified sequences, movement patterns, or predefined speeds. Thus, the subjects could perform the tasks in an individual and natural manner. Our sensor system was fixed on top of the jaws of an electromechanical goniometer (Noraxon; Scottsdale, AZ, USA), which was used for reference angle measurements, and attached laterally to the distal thigh and proximal shank with sticking plaster. Correct positioning of the goniometer was verified by active and passive flexion and extension of the knee joint. Subjects were asked whether the sensor positioning felt comfortable to ensure minimal influence on normal knee motion. Recordings were made using a wireless Noraxon system (Telemyo 2400T G2) at a sampling rate of 1500 Hz. The sensors were fixed to the right limb of healthy subjects and to the limb of the MS patients that was most affected by spastic paresis. To compare the activity classification and step detection with a commercially available system, an activPAL activity monitor (Pal Technologies, Glasgow, UK) was applied to the same limb. The sessions were recorded using a video camera.

**Table 2:** Trial protocol tasks.

No.	Exercise	Expected Activities
(I)	Sensor application	-
(II)	Sensor calibration and synchronization	standing
(III)	Sensor familiarization	sitting
(IV)	Maximum active knee flexion and extension in sitting, standing and lying posture	sitting; standing and lying
(V)	Transitions between postures	standing ↔ sitting; standing ↔ lying
(VI)	Walking standardized paths marked on the ground	walking
(VII)	25ft walk test	walking
(VIII)	Sitting and resting	sitting
(IX)	Eating a snack	sitting → walking → standing and opening a cupboard → walking → sitting while eating → walking → standing and washing hands → walking → sitting
(X)	Opening a window	sitting → walking → standing and opening window → walking → sitting
(XI)	Watching TV	sitting
(XII)	Interview	sitting → walking → standing → sitting
(XIII)	Maximum active knee flexion and extension	lying; sitting; standing
(XIV)	Sensor removal	-

The trial protocol consisted of sensor application (I) sensor calibration and synchronization; (II), sensor familiarization while sitting; (III), and repetition of maximum knee flexion and extension in different positions; (IV) (sitting, standing, and lying) to acquire the maximum active knee joint range of motion. Afterward, transitions between standing, sitting, and lying positions were recorded repeatedly; (V). The subjects were asked to walk standardized paths multiple times; (VI) along a square with diagonals that were marked on the ground, at a self-selected speed and with changes in walking directions. The subjects also performed the following activities: a 25-ft walking test; (VII), sitting and resting; (VIII), eating a snack; (IX), walking and standing while opening a window; (X), and sitting and watching TV; (XI). Finally, a short interview was conducted (XII), and the maximum active knee flexion and extension in different postures; (XIII) were repeated for a second time. The sensor system was removed afterward (XIV).

Tasks (VIII)–(XII) are included as selected activities of daily living. They are supposed to focus the subject on a different task and distract them from the activities our algorithm can classify. Table 2 lists the exercises and expected activities in sequential order.

The video recording was annotated by two independent researchers at a sampling rate of 2 Hz to generate ground truth data for activity classification and step counting. The goniometric knee angle was resampled at 50 Hz to generate ground truth data that matches our system’s sampling rate. Ambulatory behavior was divided into four classes: lying; sitting; standing; and walking. Single steps from the considered leg were counted. The annotations showed slight differences at posture transitions resulting from the researchers’ subjective view of the beginning and ending of activities. Therefore; the activity was defined as ground truth for a sample only if both researchers annotated the same activity class. Otherwise; the activity was undefined for that sample. The steps counted by the two researchers were averaged to reduce counting errors and subjective differences; for example weight transfer steps in the beginning or ending of the walking phases. The activity classifications from our algorithm and from the activPAL system were resampled at 2 Hz to create confusion matrices with the annotation data. The maximum range of motion exercises at the beginning and end of each trial were excluded from the classifier performance evaluation. Excluding one complete measurement; we recorded and annotated 1671 steps with 12.4 min of lying; 337.4 min of sitting; 29.3 min of standing; 37.3 min of walking; and 7.8 min of unknown activity for the MS patients; and 1959 steps; 14.8 min of lying; 123.7 min of sitting; 31.4 min of standing; 40.7 min of walking; and 5.5 min of unknown activity for the healthy subjects.

#### 2.3.1. Excluded Data

We excluded two measurements from the activPAL system (one faulty activity classification, one broken sensor) and two measurements from our system (one faulty classification, one disrupted measurement because of a corrupted SD card file system). A camera failure led to the exclusion of one complete measurement of the classifier validation. We also had to completely exclude the data of three subjects for knee angle validation because of technical problems with the reference goniometer during data acquisition. Recording was stopped prior to the second range of motion exercises for two subjects because of connectivity problems between the wireless Noraxon goniometer system and the PC. Three patients were unable to perform single flexion/extension tasks at the end of the trial protocol due to muscle fatigue after previous exercises.

#### 2.3.2. Statistical Analysis

The performance of both systems’ classification algorithms, including the chronological sequence of classifications, was assessed by creating confusion matrices for each system and calculating the kappa coefficient (κ) as well as the overall accuracy (OAA) [41,42]. OAA and κ values were compared using the Wilcoxon test calculated using IBM SPSS Statistics 20 (IBM Corp.; Armonk, NY, USA). Additionally, precision, sensitivity (recall), specificity, and accuracy were calculated for each activity class [43]. We also created box plots and Bland-Altman diagrams of the summarized durations of each activity class and step count. Range of motion exercises at the beginning and end of the trial protocol, as well as two recording periods consisting of ten consecutive steps that were randomly selected from the walking exercise, were used for knee angle measurement validation. Timestamps were synchronized manually and data window sizes for comparison were matched between the reference goniometer and the calculated knee angle. The root mean square error (RMSE) and Pearson’s correlation coefficient (PCC) were calculated to assess our system’s accuracy.

## 3. Results

### 3.1. Activity Classification and Step Counting

Table 3 shows the performance of each classifier in the confusion matrices. The two manual video annotations are matched in Table 3a, and show very high inter-observer agreement (>97.7%) for the activity classes lying, sitting, and walking in both groups. However, the agreement for standing activity was lower (>86%) between researchers 1 and 2 in both groups due to confusion between standing and walking activities.

The classifications by our system and by the activPAL system are matched to the merged video annotation in Table 3b,c, respectively. Our algorithm showed significantly higher classification performance (OAA = 95.2% (SD = 0.5%); κ = 0.921 (SD = 0.007)) than the activPAL system (OAA = 93.9% (SD = 0.9%); κ= 0.889 (SD = 0.019)) for healthy subjects (p ≤ 0.018). For MS-patients both systems show comparable performance (our system: OAA = 96.5% (SD = 0.9%); κ = 0.901 (SD = 0.002) *vs*. activPAL: OAA = 96.7% (SD = 0.6%); κ = 0.890 (SD = 0.018); p ≥ 0.11). Our system could distinguish between the activity classes sitting and lying with a good strength of agreement. Both sensor systems showed the highest level of confusion between walking and standing activities. General performance parameters are calculated for both systems in Table 4. Our algorithm showed high accuracy (>0.98), precision (>0.89), sensitivity (>0.92), and specificity (>0.98) for all activity classes.

**Figure 3:**
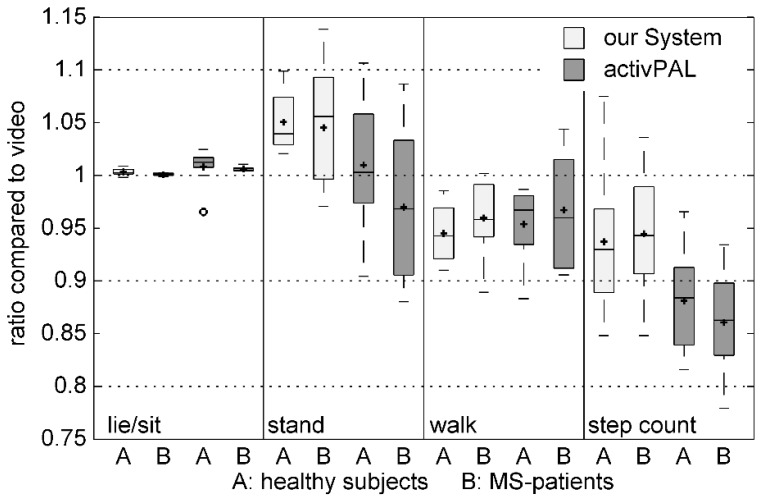
Differences between summarized activity durations from our System/activPAL compared to ground truth video data. (A: healthy subjects, B: MS patients, light gray: our system, dark gray: activPAL, rings: outliers).

**Figure 4:**
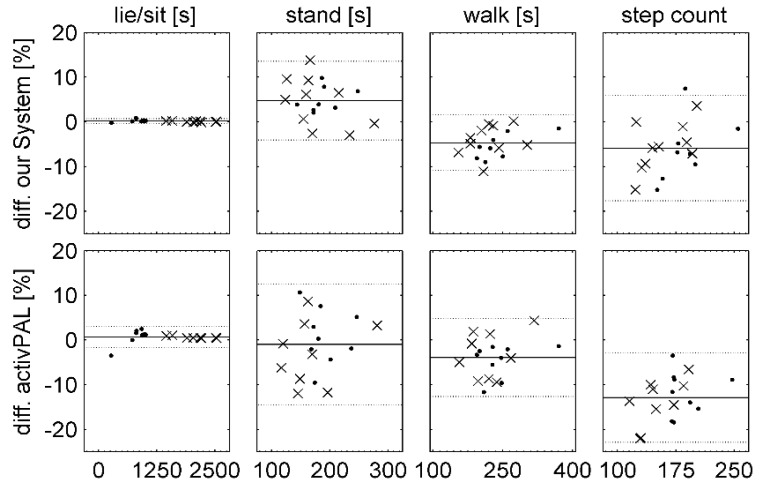
Difference of summarized activity time for our algorithm and the activPAL system compared to video data, shown as Bland-Altman plot (o: healthy subjects, X: MS patients, black line: mean error, gray line: mean error ± 1.96 SD).

The boxplots in Figure 3 show the total durations for each activity class and the step count from both monitoring systems normalized to ground truth data without regard to chronological sequence. Outliers and extreme values were excluded from further analysis. The Bland-Altman plots in Figure 4 demonstrate the percentage difference between the recognized activity duration and the step count data from the video annotation data. Mean and SD values are listed in Table 5. While the total walking time and summarized step count were underestimated by both systems, our algorithm showed a smaller deviation from the ground truth data for step counts.

**Table 3:** Confusion matrices (values in %) (N/D: not defined, e.g., transition between categories) (**a**) manual video annotation researcher 1 compared to researcher 2; (**b**) our presented algorithm compared to the merged video annotation; (**c**) activPAL system compared to the merged video annotation.

				**Video Annotation-Researcher 2**				
				**Lie**	**Sit**	**Stand**	**Walk**	**N/D**
(**a**)	Video Annotation - Researcher 1	A (n = 10)	Lie	**99.50**	0.50			
			Sit	0.11	**99.08**	0.02	0.01	0.78
			Stand		0.31	**90.62**	5.34	3.73
			Walk			1.88	**97.70**	0.42
		B (n = 10)	Lie	**99.07**	0.93			
			Sit	0.20	**99.39**		0.01	0.41
			Stand		0.32	**85.95**	8.13	5.60
			Walk			1.77	**98.01**	0.22
				**Our Algorithm**				
				**Lie**	**Sit**	**Stand**	**Walk**	**N/D**
(**b**)	Merged Video Annotation (ground truth)	A (n = 8)	Lie	**96.94**	3.06			
			Sit	0.07	**99.88**	0.01		0.04
			Stand		0.82	**96.45**	2.73	
			Walk		0.70	6.37	**92.87**	0.05
		B (n = 10)	Lie	**92.68**	7.32			
			Sit	0.11	**99.71**	0.12	0.01	0.04
			Stand		2.08	**92.63**	5.21	0.09
			Walk		0.56	7.57	**91.88**	
				**activPAL**				
				**Lie/sit**		**Stand**	**Walk**	**N/D**
(**c**)	Merged Video Annotation (ground truth)	A (n = 9)	Lie	**100.00**				
			Sit	**99.70**		0.30	0.01	
			Stand	5.22		**89.24**	5.54	
			Walk	0.83		7.87	**91.30**	
		B (n = 9)	Lie	**100.00**				
			Sit	**99.98**		0.02		
			Stand	6.78		**87.36**	5.86	
			Walk	0.32		7.20	**92.48**	

**Table 4:** Classifier performance parameters for each predicted activity class compared to video annotation.

		Our Algorithm		activPAL	
		A	B	A	B
**Lying**	Precision	0.99	0.97	not applicable, distinction between lying and sitting posture is not possible due to functionality	
	Sensitivity	0.97	0.93		
	Specificity	1	1		
	Accuracy	1	1		
**Sitting**	Precision	0.99	0.99		
	Sensitivity	1	1		
	Specificity	0.99	0.98		
	Accuracy	0.99	0.99		
**Lying + Sitting**	Precision	1	1	0.99	0.99
	Sensitivity	1	1	1	1
	Specificity	0.99	0.99	0.97	0.97
	Accuracy	1	1	0.99	0.99
**Standing**	Precision	0.92	0.89	0.89	0.90
	Sensitivity	0.96	0.93	0.89	0.87
	Specificity	0.98	0.99	0.98	0.99
	Accuracy	0.98	0.99	0.97	0.98
**Walking**	Precision	0.98	0.96	0.96	0.95
	Sensitivity	0.93	0.92	0.91	0.92
	Specificity	0.99	1	0.99	1
	Accuracy	0.98	0.99	0.97	0.99

**Table 5:** Summarized activity durations, difference compared to video recording.

	Difference [%] Mean (SD)	
	Our System	activPAL
Lie/Sit	0.18 (0.27)	0.70 (1.21)
Stand	4.75 (4.50)	−1.02 (6.91)
Walk	−4.68 (3.17)	−3.95 (4.44)
Step Count	−5.87 (6.02)	−12.92 (5.11)

### 3.2. Knee Angle Measurement

Table 6 shows the results from the knee angle measurements. The RMSE and PCC were calculated between measured (mechanical goniometer) and time-synchronized data recorded by our system for each motion exercise. Time window durations differed between subjects depending on the task execution speed. All exercises showed a RMSE of less than 5° compared with the ground truth data.

**Table 6:** Comparison of measured (goniometer) and predicted (our system) knee angles calculated as root mean square error (RMSE) in angular degree and Pearson correlation coefficient (PCC) for each sequence.

			Quality of Knee Angle Measurement	
		n	RMSE [°] Mean (SD)	PCC Mean (SD)
**Activity**	**ROM lying**	34	4.86 (1.97)	0.999 (0.000)
	**ROM sitting**	33	2.91 (1.09)	0.999 (0.001)
	**ROM Standing**	32	2.37 (0.78)	0.999 (0.001)
	**Walking**	36	3.63 (1.23)	0.975 (0.026)

## 4. Discussion

The reliable, long-term measurement of physical activity and joint motion can be a helpful instrument for the assessment of rehabilitation status and functional outcome in clinical and domestic environments [28]. The widespread occurrence of walking impairment caused by different neurologic or orthopedic disorders demonstrates that there is a need for a robust and universally applicable measurement system. The aim of the present study was to describe a cost-efficient sensor hardware and software system for the measurement of ambulatory behavior and knee joint motion in a home care environment. The new system was evaluated against video recording data and compared to commercially available activPAL sensors. Healthy subjects and patients suffering from MS performed a test protocol simulating daily living activities. The huge differences in movement and gait performance between healthy subjects and MS patients were used to stress test the system. Gait was impaired in some patients who needed walking aids (e.g., crouches, walking sticks, or walking frames) to complete our protocol. A sensor system for long-term measurements in a less controlled home care environment should nonetheless be able to deliver valid and meaningful data. Furthermore, it should be unobtrusive, not restricted to certain prepared rooms and independent of separate devices for power supply and data recording with a capability of recording data for multiple days. Therefore, our minimalistic sensor system is based on currently available, low-power-consuming electronic components without real-time evaluation capabilities. We did not use machine learning methods, e.g., neural networks or support vector machines, to solve the tasks because the algorithm needed to be computationally simple to be integrated into the utilized microprocessor for real-time processing at a later development stage.

The uniaxial activPAL sensor was defined as the reference for activity classification performance: however, a direct comparison to that system is difficult because of its much simpler design. Because it is worn on a single location, it groups sitting and lying activities, which limits its functionality. ActivPal’s advantages are its much smaller size and longer battery runtime. It has been validated in healthy subjects [44,45,46,47], and it is an accepted monitor for the assessment of physical activity. Because of our more complex design using accelerometers and gyroscopes on two body segments, which was selectively developed for patients with neuromuscular disorders, our system should at least reach or exceed a comparable accuracy.

Our system showed significantly higher classification performance for healthy subjects and comparable performance for MS patients. Additionally, our system showed a good distinction between the sedentary postures of lying and sitting, whereas the activPAL system does not provide this function. Other studies that have also used video-based evaluation protocols and activPAL monitors have reported similar classification accuracies [44,45]. Both systems underestimated step counts compared with video annotation. ActivPAL showed an approximately a two-fold higher mean deviation. Several studies reported higher accuracy for step counting using video recording data compared with activPAL in a treadmill/outdoor walking protocol [46] and a treadmill-only protocol [47]. The main reason for this difference might be the simulation of activities of the home environment, including several changes of walking direction in the present study, while most studies have used a treadmill at different walking speeds. Furthermore, the measurement of the knee joint flexion-extension angle showed lower accuracy compared with optical motion analysis systems used in gait laboratories [48]. However, averaged RMSEs were within 5°, which, according to the American Medical Association, is a clinically accepted mean error limit for reliable joint angle measurements in the evaluation of movement impairments [49]. Because our system and the reference goniometer were rigidly connected, any influences on knee joint motion were measured by both systems equally and therefore should not affect our performance calculations. The influences on absolute knee angle accuracy depending on soft tissue movement or missalignment should be considered by employing a more accurate 3-dimensional motion capturing system in future studies.

There are some technical limitations resulting from the low cost design of the presented hardware and the moderate complexity of the proposed algorithms. First, the knee joint, with all its degrees of freedom, is simplified as a hinge joint by our algorithm. Varus-valgus movements and internal-external rotations, as well as all translations [50,51,52], are ignored and superimposed onto the calculated flexion-extension joint angle. This angle is best approximated with the sensor’s *z*-axes aligned to the knee joint’s main axis of rotation. As the axis of rotation is not constant throughout movement of the anatomic knee joint, perfect alignment of sensor axes may never be achieved for the whole range of motion. Additionally, there are subject-dependent movement artifacts caused by skin and tissue motion, which affect all externally applied sensors [53,54].

Second, if the sensor is tilted from the sagittal plane, for example, during sitting with legs crossed or lying on one’s side, the error for the joint angle calculation from the accelerometer data will increase. Tilting the sensor from the sagittal plane was noticeable in our range of motion exercise, where the lying exercise showed an RMSE that was almost twice those of the sitting or standing exercises. The reason for this difference might be that the subjects’ legs were slightly rotated toward the outside when they were lying comfortably on their backs.

Another limitation is the irregular distribution of the activity durations throughout our tests. Although the exercises should simulate the activities of daily living; the distribution of activity classes is not related to typical activity profiles in a home care environment [55,56]. The differences between both manual video annotations probably resulted from subjectivity when viewing the beginning and ending of activities. The visual distinction between standing and walking during slow transitions was particularly difficult and should be considered in further related work.

## 5. Conclusions

The present article describes the development of hardware and a software algorithm for a simplified ambulatory behavior monitoring system that is also capable of measuring the knee joint angle in a simulated home care environment without the need for expensive and complex laboratory equipment. The hardware of our activity monitor consists of few components and, therefore is cost-efficient. The categorization of different activity classes in chronological sequence and summarized in time over the measuring periods showed comparable results to a clinically accepted, commercially available physical activity monitor. The step detection algorithm delivered good accuracy compared with the reference system. The algorithm can be programmed directly into modern, low-current microprocessors because of its simplicity. In terms of the overall complexity and total costs of the sensor system, we conclude that the accuracy is sufficient for long-term monitoring of ambulatory behavior and range of motion of the knee joint in healthy subjects and patients with impaired gait performance. Thus, the new system can be considered to be a valuable clinical tool for monitoring the effectiveness of therapy. In future, the battery runtime has to be extended by using a larger battery or improving the sensors firmware to exceed a minimum recording time of seven days without recharging the system.
